# Exploring the Genomics of Marnaviridae Family: Identification, Characterization, and Taxonomic Implications

**DOI:** 10.1155/ijm/7188239

**Published:** 2026-04-20

**Authors:** Mikaela Renata Funada Barbosa, Endrya do Socorro Foro Ramos, Fabiola Villanova, Renan Lourenço Oliveira Silva, Suzi Cristina Garcia, Ronalda Silva de Araújo, Maria Cássia Mendes-Correa, Tania Regina Tozetto-Mendoza, Wen Zhang, Ramendra Pati Pandey, Adriana Luchs, Maria Inês Zanoli Sato, Antonio Charlys da Costa, Elcio Leal

**Affiliations:** ^1^ Department of Environmental Analysis, Environmental Company of the São Paulo State (CETESB), São Paulo, Brazil; ^2^ Viral Diversity Laboratory, Institute of Biological Sciences, Federal University of Pará, Belém, Pará, Brazil, ufpa.br; ^3^ Department of Infectious and Parasitic Diseases, Faculty of Medicine, University of São Paulo, São Paulo, Brazil, usp.br; ^4^ Department of Laboratory Medicine, School of Medicine, Jiangsu University, Zhenjiang, China, ujs.edu.cn; ^5^ School of Biosciences and Bioengineering, D Y Patil International University, Akurdi, Pune, 411044, Maharashtra, India; ^6^ Institute Adolfo Lutz, São Paulo, Brazil

**Keywords:** freshwater, Marnaviridae, metagenomic, phylogenetics, virome

## Abstract

In this study, we characterized sequences similar to Marnaviridae obtained from water samples in the state of São Paulo, Brazil. Sixteen complete or nearly complete genomes were determined, all of them positive‐sense single‐stranded RNA, with lengths between 7074 and 10,198 base pairs, containing one or two open reading frames (ORFs). The amino acid sequences derived from the ORFs showed similarity and protein domains typical of the Marnaviridae family. Phylogenetic analysis based on RNA‐dependent RNA polymerase (RdRp) revealed clusters closely related to viruses that have not yet been classified by the International Committee on Taxonomy of Viruses (ICTV). Some sequences showed proximity to established genera such as *Salicharnavirus*, *Locarnavirus*, and *Labynarvirus*, while others formed three distinct clades, suggesting the presence of new genera. Furthermore, one sequence displayed an RdRp identity of less than 90% and a capsid identity of less than 75%, indicating that it represents a novel species related to Marnaviridae. These findings expand current knowledge of Marnaviridae diversity, contributing to a better understanding of evolutionary relationships and emphasizing the need for taxonomic reorganization.

## 1. Introduction

Marine RNA viruses are abundant and play an important role in aquatic ecosystems, modulating populations including bacterial and algal populations and participating in nutrient cycling [1, 2]. The first marine RNA virus, Heterosigma akashiwo RNA virus, which infects phytoplankton, was discovered in 2003 [3, 4].

Since then, other viruses have been isolated infecting different marine unicellular eukaryotes (protists) including Rhizosolenia setigera RNA virus 01 in 2004 (infects Rhizosolenia setigera) [5], Aurantiochytrium single‐stranded RNA virus 01 in 2005 (infects Aurantiochytrium) [6], Chaetoceros tenuissimus RNA virus 01 in 2008 (infects Chaetoceros tenuissimus) [7], Chaetoceros socialis forma radians RNA virus 1 in 2009 (infects Chaetoceros socialis *f*. radians) [8], and Asterionellopsis glacialis RNA virus in 2012 (infects Asterionellopsis glacialis) [9], in Japan. Other viruses were discovered through metagenomic studies performed on marine and freshwater samples [10–13].

All of these viruses share common features, including the replication block helicase (Hel), 3c‐like protease (Pro), and RNA‐dependent polymerase (RdRP) characteristic of members of the order *Picornavirales*, resulting in the creation of the Marnaviridae family [14].

Members of Marnaviridae have linear genomes of positive‐sense ribonucleic acid (+ssRNA) with mono or dicistronic organization, and both encode nonstructural proteins in the 5′ region (RdRP, Hel, and Pro) and structural proteins in the 3′ region (VP1 to VP4) [15].

According to the International Committee on Taxonomy of Viruses (ICTV), the family contains seven genera: *Bacillarnavirus*, *Kusarnavirus*, *Labyrnavirus*, *Locarnavirus*, *Marnavirus*, *Salisharnavirus*, and *Sogarnavirus*, defined based on phylogenetic analysis of the amino acid sequences of the RdRP domain. Within genera, species are defined based on comparison of pairwise amino acid identity, with viruses of a new species sharing less than 90% identity in the RdRP and 75% in the capsid [15].

Based on these criteria and advances in omics, the number of unclassified viral sequences available in public databases has increased. A single study reported the identification of 1445 RNA viruses with high genetic diversity at the family and genus level, including within Marnaviridae [16]. This highlights the need to reorganize the current taxonomy to reflect these new data.

In this study, we describe the characterization of 16 genomes (9 complete) related to Marnaviridae obtained from water from different points located in the state of São Paulo. Six sequences were related to three known genera, *Salicharnavirus*, *Locarnavirus*, and *Labynarvirus*, and another 10 established three probable new genera and one new species.

## 2. Materials and Methods

### 2.1. Water Sample Collection Locations

Monitoring of viruses in surface freshwater was conducted at 12 sites in the Alto Tietê Basin, in the State of São Paulo, Brazil. However, the members of Marnaviridae described in this study were detected in two reservoirs, Guarapiranga (−23.6741, −46.7277) and Taiaçupeba (−23.5786, −46.2780), and in two rivers, Tietê (−23.5216, −46.6311 and −23.4547, −46.9100) and Pinheiros (−23.5311, −46.7483). Water sample collection and processing were carried out by the laboratories of the Department of Environmental Analysis of CETESB (Environmental Agency of the State of São Paulo). Briefly, 10 L of reservoir water and 1.5 L of river water were collected biweekly, between August 2022 and July 2023. Samples were transported on ice, stored at 4°C–8°C, and processed within 24 h of collection [17, 18].

### 2.2. Preprocessing of Water Samples

River water samples (250 mL) were centrifuged at 3500 × g for 30 min, and the supernatant was concentrated by ultrafiltration using two Centricon Plus‐70 filters (10 kDa) (Merck Millipore, MA, USA) per sample. Filters were centrifuged at 3000 × g for 20 min until the concentrate volumes were reduced to approximately 300 μL. Concentrates were recovered by centrifugation at 1000 × g for 2 min.

Reservoir water samples (10 L) were processed by hollow‐fiber ultrafiltration (HFUF) using a dead‐end ultrafiltration (DEUF) system, as described by Smith and Hill (2009). Briefly, HdF100 S hemodialysis filters (Fresenius Medical Care, Bad Homburg, Germany) were pretreated with 1 L of 0.01% sodium polyphosphate (NaPP) solution (Sigma‐Aldrich, St. Louis, MO, USA), and after sample filtration, viruses were eluted using 500 mL of elution buffer containing 0.01% Tween 80, 0.01% NaPP, and 0.001% Antifoam Y‐30 emulsion (Sigma‐Aldrich, St. Louis, MO, USA). For the secondary concentration, viruses were precipitated with polyethylene glycol (PEG), following the protocol described by Shulman et al. (2006). The primary concentrate was mixed with 40 g of PEG 6000 and 8.75 g of NaCl. Samples (pH 7.2–7.4) were gently mixed on a magnetic stirrer and incubated at 4°C for a minimum of 16 h. After overnight incubation, samples were centrifuged at 4750 × g for 1 h. The supernatant was carefully removed, and the pellet was suspended in the residual volume. All sample concentrates were aliquoted and stored at −80°C until further processing.

Concentrated samples, pooled monthly and by site, were homogenized with Vertrel XF (DuPont) in a volume corresponding to 10% of the sample volume. After homogenization and centrifugation (7500 × g for 15 min), the supernatant was filtered through Ultrafree‐MC HV 0.45‐μm sterile filters (8,000 rpm for 5 min) to reduce nonviral nucleic acids. The resulting filtrate was subsequently treated with DNase (Thermo Fisher Scientific, Waltham, MA, USA). Pretreated samples were then subjected to automated DNA/RNA extraction using the Extracta 16 platform (Loccus Biotecnologia, Brazil) and magnetic beads (Promega), according to the manufacturer’s instructions. Alternatively, concentrated samples were treated with Vertrel and extracted using the AllPrep PowerViral DNA/RNA kit (Qiagen).

### 2.3. Extraction, cDNA Synthesis, Library Preparation, Sequencing, and Quality Control

Samples were processed to eliminate potential nonviral RNA and DNA contamination through enzymatic treatment with TURBO DNase (Thermo Fisher Scientific, Waltham, MA, USA). Nucleic acids (DNA/RNA) were extracted using the Quick‐DNA/RNA Pathogen MagBead kit (Zymo Research), following the manufacturer’s instructions. Extracted nucleic acids were used at their original concentrations, with viral DNA and RNA sequences subjected to reverse transcription using SuperScript IV reverse transcriptase (Thermo Fisher) and 100 pmol of a random decamer primer. This was followed by a single round of second‐strand DNA synthesis with Klenow fragment polymerase (New England BioLabs). Libraries were then constructed using the Nextera XT DNA kit.

Libraries were prepared using the Illumina Sample Preparation Kit and sequenced on the Illumina NovaSeq 6000 platform with 250‐bp paired‐end reads and dual barcoding. All procedures were conducted under strict precautions to prevent cross‐contamination and nucleic acid degradation. Aerosol‐resistant filter tips were used to minimize contamination risk, and all consumables (e.g., microcentrifuge tubes and tips) in direct contact with nucleic acids were DNase‐ and RNase‐free. Samples were dissolved in DEPC‐treated water supplemented with RNase inhibitors. Blank controls consisted of sterile ddH_2_O, prepared and processed under identical experimental conditions. Quality assessment was performed using agarose gel electrophoresis and the Agilent Bioanalyzer 2100. Sequencing of the control pool on the NovaSeq platform yielded only a minimal number of reads. Data were initially processed using the “virus discovery” pipeline of the Department of Laboratory Medicine, School of Medicine, Jiangsu University, Zhenjiang, Jiangsu, China, on supercomputers [19].

### 2.4. Contig Coverage Analysis

Viral contigs were analyzed with CheckV v1.5 to estimate completeness (0%–100%) and classify viral genome quality into five levels: complete, high (> 90%), medium (50%–90%), low (0%–50%), or indeterminate, based on comparison to complete viral genomes from a FASTA file [20].

### 2.5. Taxonomic Assignment of Contigs and Annotation

Complete or nearly complete nucleotide sequences were searched against the NCBI nonredundant (NR) protein database using BLASTx to identify the best hits. Results with the highest percentage of identity and an E‐value < 1E‐5, relative to sequences already deposited in GenBank, were selected [21].

### 2.6. Genome Annotation

Open reading frames (ORFs) in the genomes were identified using ORFfinder (https://www.ncbi.nlm.nih.gov/orffinder/). Conserved protein domains and motifs were predicted using the CDD Conserved Domain Database [22], Motif Finder (https://www.genome.jp/tools/motif/, accessed April 1, 2025), Palmscan [23], and LucaProt [24].

### 2.7. Alignment and Phylogenetic Analysis

Amino acid sequences from this study were aligned with members of Marnaviridae and other related viruses using the MUSCLE algorithm [25] implemented in the UGENE platform [26]. Maximum likelihood (ML) phylogenetic trees were inferred with IQ‐TREE software [27] under the best‐fit model. Node support was assessed with 1.000 ultrafast bootstrap replicates, and the resulting trees were visualized using FigTree v5 (//tree.bio.ed.ac.uk/software/figtree).

### 2.8. Evolutionary Distances

Genetic distances and their standard errors were calculated using MEGA v12, applying the ML model with 1.000 bootstrap replications to assess the robustness of the estimates. This approach provided statistical support for the calculated distances both within and between clades [28].

Sequence similarity was estimated using the SDT program v1.2 [29], which computes pairwise similarity alignments with MUSCLE algorithms. Identity scores for each pair were then used by PHYLIP’s NEIGHBOR component to generate a rooted neighbor‐joining phylogenetic tree, organizing sequences according to their evolutionary relatedness. Results were displayed as a frequency distribution of pairwise identities in a graphical interface.

## 3. Result and Discussion

### 3.1. Analysis of Nucleotide Sequences Identified in Freshwater

Next‐generation sequencing (NGS) identified 16 contigs from six sequencing libraries derived from freshwater samples collected in the Guarapiranga Reservoir, Tietê River, and Pinheiros River, all located in the metropolitan area of São Paulo State, Brazil. These contigs were analyzed against the NCBI NR database using BLASTx. All contigs showed associations with viruses not yet classified by the ICTV [30–32], with the closest similarities found within the Marnaviridae family (Table 1).

**TABLE 1:** Description of contigs and comparison of BlastX for identification in water samples in Brazil.

Library code	Contig_ID	Length (bp)	GC content	Genome completeness	Best‐hit BLASTx	Identity	Coverage	E‐values
S5_L001	SP_11226	8670 nt	40.90%	CG ‐ (95.84%)	XCM67885	96.64%	60%	0.0
S3_L001	SP_44182	7711 nt	41.65%	PG ‐ (85.47%)	XCM67885	80.80%	61%	0.0
S5_L001	SP_34331	8091 nt	41.10%	PG ‐ (89.58%)	XCM67885	81.53%	60%	0.0
S6_L001	SP_40484	7494 nt	42.07%	PG ‐ (83.00%)	XCM67885	79.00%	56%	0.0
S5_L001	SP_24176	10198 nt	45.03%	CG ‐ (98.06%)	WVR21149	100%	57%	0.0
S22_L001	SP_44506	10021 nt	44.99%	CG ‐ (97.65%)	WVR21149	99.95%	59%	0.0
S3_L001	SP_83441	10144 nt	45.08%	CG ‐ (97.93%)	WVR21149	100%	57%	0.0
S22_L002	SP_10981	7431 nt	45.45%	PG ‐ (72.48%)	WVR21149	100%	51%	0.0
S3_L001	SP_46102	7207 nt	42.12%	PG ‐ (77.0%)	XCM68032	58.05%	78%	0.0
S39_L002	SP_44658	8465 nt	44.10%	CG ‐ (96.47%)	XCM68032	60.92%	56%	0.0
S31_L002	SP_28427	9352 nt	40.68%	CG ‐ (100%)	XHA88067	39.13%	81%	0.0
S31_L001	SP_96915	7462 nt	40.21%	PG ‐ (83.17%)	XHA88067	41.05%	79%	0.0
S3_L001	SP_71772	7074 nt	40.45%	PG‐ (80.13%)	XCM68328	98.55%	73%	0.0
S3_L001	SP_36073	9601 nt	45.25%	CG‐ (100%)	WPR17964	96.07%	89%	0.0
S3_L001	SP_31066	9398 nt	42.40%	CG‐ (100%)	XCM67841	53.17%	67%	0.0
S5_L001	SP_21627	9653 nt	42.43%	CG‐ (100%)	XCM67841	53.17%	65%	0.0

*Note:* ID: contig identification.

Abbreviations: bp: base pairs, CG: complete genome, PG: partial genome.

### 3.2. Organization of the Genome

All genomes are ssRNA, with lengths ranging from 7.074 to 10.198 bp and GC content between 40.45% and 45.03% (Table 1). Genome analysis predicted either a single ORF: SP_36073, SP_28427, and SP_96915 (Figure 1(a)) or two ORFs: SP_11226, SP_34331, SP_40484, SP_44182, SP_24176, SP_44506, SP_10981, SP_83441, SP_46102, SP_44658, SP_71772, SP_31060, and SP_21627) (Figure 1(b)) all oriented from 5′ to 3’.

FIGURE 1Genome organization of viruses identified in water samples. (a) Monocistronic genome map. (b) Dicistronic genome map. Both contain conserved domains, including RNA helicase (yellow), peptidase_C3 (blue), RNA‐dependent RNA polymerase (green), rhv‐like domains (orange), VP4 dicistronic (red), and CRPV (yellow).

**Figure:**
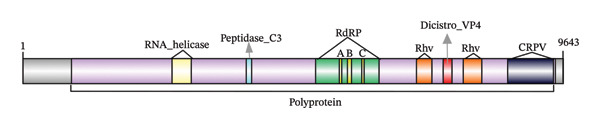
(a)

**Figure:**
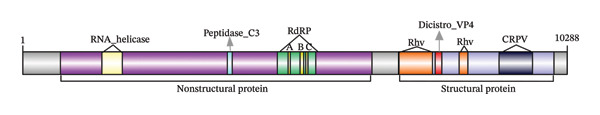
(b)

Both monocistronic and dicistronic genomes encode the nonstructural proteins Hel, Pro, and RdRP, as well as the structural proteins VP2, VP4, VP3, and VP1. These proteins are essential for viral replication and capsid assembly. This organization is typical of *Picornavirales* members [14] (Table S1).

### 3.3. RNA Helicase Domain

The putative RNA‐Hel domain (PF00910) was predicted in all sequences except SP_10981. In this region, conserved motifs similar to Walker A (GxxGxGKS/T), B (Qx 5 DD), and C (KKx 4 Px 5 NSN) were identified [33–35].

The sequences analyzed here showed amino acid substitutions compared to the consensus. In the putative Walker A motif, the consensus sequence GxxG/SGxKS/T displayed a substitution of glycine (G) by serine (S).

In Walker B, the consensus sequence was Q/I/S/T *x*_5_ D/ND, with substitutions of glutamine (Q) by isoleucine (I), serine (S), and threonine (T), and aspartate (D) by asparagine (N) and alanine (A), the latter representing a highly conserved residue.

Walker C displayed the consensus K/E/G/A K/M *x*_4_ P/F/S/H/Q/L *x*_5_ T/D/S/TN, which was less conserved. This amino acid variability is consistent with patterns observed in other viral families within the order, including Marnaviridae (Table S2).

### 3.4. Protease Domain

The Pro domain (PF00548) was identified in eight sequences. Within this domain, the cysteine protease motif GxCG and the substrate‐binding motif GXHXXG were detected [36], corresponding to GLCG–GLHLGG (SP_11226, SP_44182, SP_34331, SP_40484) and GDCG–GIHIAG (SP_24176, SP_44506, SP_83441, SP_10981) in the amino acid sequences analyzed (Table S3). The presence of distinct motifs may indicate functional or evolutionary divergence among the sequences, thereby contributing to the understanding of the molecular and functional diversity of cysteine proteases.

### 3.5. RdRP Domain

The RdRP domain is essential for elucidating the evolutionary patterns of RNA viruses. In the sequences identified in this study, the RdRp region (PF00680) ranged from 307 to 400 residues. Sequence identity values of 99.9% (SP_44182, SP_34331, SP_40484, SP_24176, SP_44506, SP_46102, SP_83441, SP_10981) and 100% (SP_11226, SP.33_44658, SP_28427, SP_96915, SP_71772, SP_36073, SP_31066, SP_21627) were obtained in LucaProt [24]. We compared the most conserved catalytic motifs: A, B, and C, with the sequences of greatest identity obtained by BLAST (Figure S1 and S2).

### 3.6. Domains in Structural Proteins

The framework region was predicted to encode the structural proteins VP2, VP4, VP3, and VP1. Conserved domains were also identified, including rhv‐like (PF00073), Dicistro VP4 (PF11492), Calici_coat (PF00915), Waikav_capsid_1 (PF12264), and CRPV (PF08762) (Table S1).

### 3.7. Phylogenetic Analysis and Genetic Distances

To assess taxonomic relationships, phylogenetic inference was conducted using the ML method with 1000 bootstrap replicates. The analysis employed amino acid sequences from the RdRp domain of 20 species comprising the Marnaviridae family, the best BLASTx matches, and representatives of the nine families within the Picornavirales order available in the NCBI database.

**FIGURE 2:**
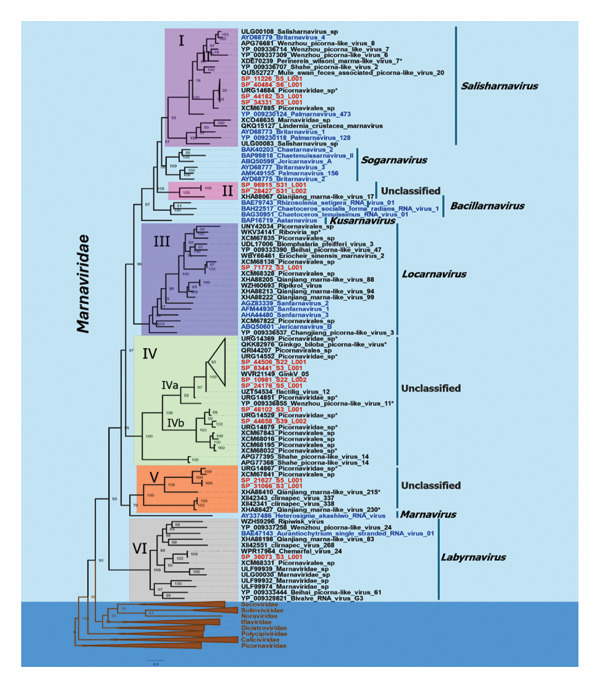
Maximum likelihood phylogenetic tree based on RdRp amino acid sequences. Viruses identified in water are named with contig and library identification (red font), members of Marnaviridae (blue font), and representative *Picornavirales* (brown font). The GenBank accession number is provided for each sequence, followed by the species or virus name. The tree was inferred with Iq‐Tree v 2.4.0 using the VT + F + R8 substitution model with 1000 bootstraps. Bootstrap values are displayed at each node of the tree.

Phylogenetic trees were also inferred using genomic sequences (Figure S3) and amino acid sequences from the region encoding structural proteins (Figure S4) for Marnaviridae. The results corroborated the RdRp motif classification, revealing that the 16 sequences clustered into six distinct clades (Figure 2).

#### 3.7.1. *Salisharnavirus*

In Clade I, four sequences (SP_11226, SP_40484, SP_44182, SP_34331) clustered with the genus *Salisharnavirus*. Pairwise comparisons of the RdRp region revealed 98.38%–99.68% identity among them and 97.40%–98.38% identity with the partial sequence of the unclassified virus URG14684. They shared 36.12%–61.07% identity with the species of *Salisharnavirus*. For the capsid, SP_11226, SP_40484, SP_44182, and SP_34331 exhibited 84.87%–99.76% identity among themselves and 33.56%–48.27% identity with *Salisharnavirus* species. Based on the demarcation criteria for new species within the family Marnaviridae (< 90% identity in RdRp and < 75% in capsid), these sequences indicate membership in the same species as URG14684.

#### 3.7.2. *Locarnavirus*

The SP_71772 sequence in Clade III clustered near members of the *Locarnavirus* genus. Comparisons with reference sequences of *Locarnavirus* revealed 44.52%–57.42% identity in the RdRp region and 32.92%–40.06% identity in the capsid. SP_71772 exhibited the highest identity (99.34%) with the partial sequence XCM68328, indicating that it corresponds to the same species, and 87.06% identity with the complete sequence XCM68138 in RdRp. In the capsid, 78.19% identity was observed with XCM68139.

#### 3.7.3. *Labyrnavirus*

SP_36073 in Clade VI clustered with *Labyrnavirus*. It exhibited 97.99% identity in the RdRp region and 97.79% identity in the capsid with WPR17964, indicating that they represent the same viral species previously designated as Chemarfal virus. In comparison with the *Labyrnavirus* species Aurantiochytrium single‐stranded RNA virus 01 (BAE47143 and YP_398835.1), SP_36073 shared 50.19% identity in RdRp and 33.20% identity in the capsid.

#### 3.7.4. Unclassified Clade

The remaining clades (II, IV, and V) are phylogenetically divergent and remain unclassified relative to the seven established genera.

Clade II contains three sequences (XHA88067, SP_96915, and SP_28427). SP_96915 and SP_28427 share 100% identity in RdRP, 57.72% with Qianjiang_marna‐like_virus_17 (XHA88067), suggesting it is a new species of Marnaviridae. In relation to other established genera in Marnaviridae, it has 40.20%–41.70% with the *Bacillarynavirus,* 40.39% with *Kusarnavirus* species, 27.27% with *Labyrnavirus* species, 35.50 to 39.34%with the *Locarnavirus* species, 30.89% with the *Marnavirus* species, 38.70%–42.59% with the *Salisharnaviruses*, and 41.91%–47.23% with the *Sogarnavirus*. Based on the capsid, SP_96915 and SP_28427 have 88.50% identity with each other, 24.30% and 36.60% with the sequence XHA88067, and 28.23%–32.57% with the genus *Sogarnavirus*.

Clade IV has 22 sequences, distributed into two subclades. Subclade IVa (SP_44506, SP_83441, SP_10981, SP_24176) shares 100% identity among themselves and with WVR21149, indicating that they represent the same viral species. Compared with closely related viruses (UZT54534, QKK82976, QRI44207, URG14552), identities ranged from 81.90% to 96.75%.

Subclade IVb (SP_46102, SP_44658): SP_46102 exhibited 95.76% identity with the closest partial sequence URG14529 in RdRp, suggesting conspecificity. SP_44658 showed 93.76% identity with URG14579 in RdRp, also indicating conspecificity. However, identities between SP_46102 and SP_44658 were 82.40% in RdRp and 52.65% in capsid, demonstrating that they correspond to distinct species. When compared with Subclade IVa, SP_46102 and SP_44658 displayed 49.85% identity in RdRp and 35.12% in capsid, highlighting substantial divergence between subclades that may reflect specific environmental or host adaptations.

Relative to established Marnaviridae genera, the six sequences exhibited identities of 30.00%–34.11% with *Bacillarnavirus*, 33.34%–35.02% with *Kusarnavirus*, 25.38%–29.01% with *Labyrnavirus*, 34.45%–38.98% with *Locarnavirus*, 28.02%–31.25% with *Marnavirus*, 28.52%–35.00% with *Salisharnavirus*, and 29.09%–33.56% with *Sogarnavirus*. These results confirm that the sequences form a distinct lineage, corroborating a previous study that proposed the new genus Ginkgornavirus within Marnaviridae, based on the grouping of WVR21149 and four additional viruses forming a separate clade [30].

Clade V comprises eight sequences, with SP_21627 and SP_31066 sharing 99.75% identity. Compared with the closest sequence XCM6784, they exhibit 99.75% and 99.32% identity in the RdRp region, and 75.59% and 81.82% identity in the Cap region, respectively. Relative to members of Marnaviridae, RdRp identity values were 29.82%–31.70% with *Bacillarynavirus*, 36.67% with *Kusarnavirus*, 28.30% with *Labyrnavirus*, 34.42%–36.30% with *Locarnavirus*, 30.77% with *Marnavirus*, 29.34%–33.08% with *Salisharnavirus*, and 30.90%–36.11% with *Sogarnavirus*. These results underscore the pronounced divergence from currently recognized Marnaviridae genera.

Evolutionary distances were analyzed for the unclassified clades, both within individual clades and between them, based on amino acid substitutions in the RNA‐dependent RNA polymerase (RdRP) region. In Clade II, the average pairwise distance was 29% (95% CI: ±2%); in Clade IV, 39% (95% CI: ±2%); and in Clade V, 47% (95% CI: ±2%). The average evolutionary distances between established Marnaviridae genera and the unclassified clades were also high: 55%–67% for Clade II, 67%–71% for Clade IV, and 67%–71% for Clade V. These values highlight a significant divergence from currently recognized Marnaviridae genera.

## 4. Discussion

The detection and characterization of nine complete genomes and seven nearly complete genomes of positive‐sense single‐stranded RNA related to the family Marnaviridae in four locations across the state of São Paulo reveal a diversity that remains largely unexplored, complementing previous studies that highlight freshwater ecosystems as potential reservoirs for virus discovery [30–32].

Genomic analyses identified common features, including conserved domains and motifs in both structural and nonstructural regions, resembling those of other unclassified Marnaviridae viruses [30–32]. Notably, the RdRp region, which is highly conserved and frequently employed in taxonomic classification [24, 37], exhibited patterns with varying levels of conservation and divergence, underscoring the genetic diversity of viruses associated with the family Marnaviridae.

Some patterns, such as Types I, III, and VI, exhibited high similarity to established genera, specifically *Salisharnavirus*, *Locarnavirus*, and *Labyrnavirus*, respectively. In contrast, patterns with lower conservation levels suggest potential evolutionary or taxonomic divergence. Phylogenetic analyses supported these observations, clustering sequences into clades corresponding to recognized genera (I, III, and VI) and into distinct clades (II, IV, and V) that do not align with any of the seven genera currently defined within the family Marnaviridae, according to the genus demarcation criteria established by the ICTV [15].

Four sequences (SP_11226, SP_40484, SP_44182, SP_34331) clustered within Clade I, related to the genus *Salisharnavirus*, and likely represent the same species as the partial sequence URG14684, previously detected in sediment samples from China [38]. Similarly, sequence SP_71772, associated with the genus *Locarnavirus*, corresponds to the XCM68328 virus, identified in water samples from the Teltow Canal in Germany [30]. In addition, SP_36073 exhibited high identity with Chemarfal virus 24 (WPR17964), related to the genus *Labyrnavirus*, described in a metagenomic study of invertebrates conducted in the United States [33].

The detection of similar sequences across geographically distinct locations suggests that many viruses not yet officially classified by the ICTV may have broad distribution ranges. Such dispersion may be linked to low host specificity or high environmental adaptability [16].

In Clade II, sequences SP_96915 and SP_28427 clustered together, showing only 57.72% identity with the closest virus in the dataset, Qianjiang marna‐like virus 17 (XHA88067), identified in China [38]. This value is well below the 90% threshold adopted by the ICTV for species demarcation within the family Marnaviridae, indicating that these sequences represent a novel viral species. These findings contribute to expanding the known diversity of Marnaviridae.

Clade IV is subdivided into two subclades, IVa and IVb. Subclade IVa (SP_44506, SP_83441, SP_10981, SP_24176) exhibited high identity with WVR21149, previously described in a freshwater study in Brazil [30]. The recurrence of virus detection across different samples and locations, specifically the Guarapiranga Reservoir and the Tietê River, suggests viral distribution in surface waters of the region, potentially reflecting local ecological adaptation or sustained circulation.

Subclade IVb grouped sequences SP_46102 and SP_44658, which correspond to distinct viral species URG14529 and URG14579, respectively, described in China [38].

In Clade V, sequences SP_21627 and SP_31066 were closely related to XCM67842, detected in water samples from the Teltow Canal, Germany [30], suggesting that these viruses may undergo adaptive events driven by local selective pressures [16].

The topology of the phylogenies corroborates the data from the pairwise comparison of the RdRp and Cap regions. Values below 50% were found when comparing sequences from Clades II, IV, and V with members representing the seven genera. This low level of identity suggests that these sequences may represent distinct evolutionary lineages, indicating the possible existence of new genera within the family Marnaviridae.

It is important to emphasize that the analysis was conducted using sequences with the highest identity available in the NCBI database. The acquisition of additional data may enable more comprehensive evolutionary analyses and the establishment of more robust demarcation criteria than those currently applied, potentially resulting in taxonomic revisions of newly identified viruses. This underscores the importance of reorganizing taxonomic criteria for the classification of novel viruses [16].

In summary, the results raise important questions about the ecological role of these viruses, the mechanisms that sustain their dispersal, and the environmental factors that influence their evolution. However, understanding these dynamics remains limited by the lack of data on the specific hosts involved, which hinders more precise inferences about their ecological interactions. Nevertheless, the genomic characterization data presented in this study contribute to improving future investigations by expanding the repertoire of known viral sequences and contextualizing their occurrence in different environments. This helps expand knowledge about the diversity and evolution of viruses within the family Marnaviridae.

## Author Contributions

Mikaela Renata Funada Barbosa: formal analysis, data curation, and conceptualization. Endrya do Socorro Foro Ramos: writing–original draft and investigation. Fabiola Villanova: formal analysis. Renan Lourenço Oliveira Silva: data curation. Suzi Cristina Garcia: data curation. Ronalda Silva de Araújo: data curation. Maria Cássia Mendes‐Correa: data curation. Tania Regina Tozetto‐Mendoza: data curation. Wen Zhang: data curation. Ramendra Pati Pandey: writing–review and editing. Adriana Luchs: data curation and conceptualization. Maria Inês Zanoli Sato: data curation and conceptualization. Antonio Charlys da Costa: data curation and conceptualization. Elcio Leal: writing–original draft, investigation, and data curation.

## Funding

This study was supported by Conselho Nacional de Desenvolvimento Científico e Tecnológico, 305,566/2025‐3; Coordenação de Aperfeiçoamento de Pessoal de Nível Superior.

## Conflicts of Interest

The authors declare no conflicts of interest.

## Supporting Information

Table S1. Summary of genomic organization: identification, length, and layout. Table S2: Conserved motifs of Helicase. Table S3. Conserved motifs of Protease. Figure S1: The RdRP motif sequences of members of the genus Marnaviridae logos generated in WebLogo 3. Figure S2: Comparison of the reasons predicted in the RdRp domain of the viruses identified with the best BLASTx hit. Motif A (purple), motif B (blue), and motif C (green) are highlighted. Symbols (circle, square, triangle, diamond, and star) indicate matches between detected motif sequences. Sequences identified in the study are shown with colored circles, except SP_44658 (triangle) and SP_46102 (diamond). BLASTx references are highlighted by colored squares, except XCM68032 (star). Figure S3: Maximum likelihood phylogeny of the structural region of Marnaviridae. Viruses identified in water samples are labeled with contig and library ID (red font), Marnaviridae members (blue font), and representative Picornavirales (brown font). The GenBank accession number is provided for each sequence, followed by the species or virus name. The tree was inferred using IQ‐TREE v2.4.0, applying the LG + F + R6 substitution model with 1000 bootstrap replicates. Bootstrap support values are displayed at each tree node. Figure S4: Maximum likelihood nucleotide phylogeny of the Marnaviridae genome. Viruses identified in water samples are labeled with contig and library ID (red font), Marnaviridae members (blue font), and representative Picornavirales (brown font). The GenBank accession number is provided for each sequence, followed by the species or virus name. The tree was inferred using IQ‐TREE v2.4.0, applying the TVM + F + R6 substitution model with 1000 bootstrap replicates. Bootstrap support values are displayed at each tree node.

## Supporting information


**Supporting Information** Additional supporting information can be found online in the Supporting Information section.

## Data Availability

Clean read data have been deposited in the SRA database (PRJNA1051083). Viral genome sequences have been deposited in the NCBI GenBank database (PX241845‐PX241860).
