# Supplementary material for: Exploring the Genomics of Marnaviridae Family: Identification, Characterization, and Taxonomic Implications
Source: Int J Microbiol. 2026 Apr 20;2026:7188239. doi: 10.1155/ijm/7188239 (PMC13093544; doi:10.1155/ijm/7188239)
Supplement: Supplementary file 1 — Supporting Information Additional supporting information can be found online in the Supporting Information section. [file IJM-2026-7188239-s001.docx]

**SUPPLEMENTAL MATERIAL**

**Exploring the genomics of *Marnaviridae* family: identification, characterization, and taxonomic implications**

**Table S1.** Summary of genomic organization: identification, length, and layout**.**

| **Library code** | **Contig_ID** | **Length (bp)** | **Genome layout+** |
| --- | --- | --- | --- |
| S5_L001 | SP_11226 | 8668nt | ORF1: Hel – Pro – RdRp1 ORF2: Rhv – Dicistro_VP4 – Calici_coat . Waikav_capsid_1 – CRPV |
| S3_L001 | SP_44182 | 7711nt | ORF1: Hel – Pro –RdRp1 ORF2: Rhv – Dicistro_VP4 – Calici_coat Waikav_capsid_1 – CRPV |
| S5_L001 | SP_34331 | 8091nt | ORF1: Hel – Pro –RdRp1 ORF2: RhV – Dicistro_VP4 – Calici_coat Waikav_capsid_1 – CRPV |
| S6_L001 | SP_40484 | 7494nt | ORF1: Hel – Pro – RdRp1 ORF2: RhV – Dicistro_VP4 – Calici_coat Waikav_capsid_1 – CRPV |
| S5_L001 | SP_24176 | 10198nt | ORF1: Hel – Pro – RdRp1 ORF2: RhV – Dicistro_VP4 – RhV |
| S22_L001 | SP_44506 | 10021nt | ORF1: Hel –Pro – RdRp1 ORF2: RhV – Dicistro_VP4 – RhV |
| S3_L001 | SP_83441 | 10144nt | ORF1: Hel –Pro – RdRp1 ORF2: RhV – Dicistro_VP4 – RhV |
| S22_L002 | SP_10981 | 7431nt | ORF1: Pro – RdRp1 ORF2: rhv – Dicistro_VP4 – Rhv |
| S3_L001 | SP_46102 | 7204nt | ORF1: Hel – RdRp1 ORF2: RhV |
| S39_L002 | SP_44658 | 8465 nt | ORF1: Hel – RdRp1 ORF2: RhV – RhV |
| S31_L002 | SP_28427 | 9352nt | ORF1: Hel –RdRp1 – RhV – Dicistro_vp4 – RhV – Calici_coat – Waikav_capsid_1 – CRPV |
| S31_L001 | SP_96915 | 7462nt | ORF1: Hel – RdRp1 – RhV – Dicistro_vp4 |
| S3_L001 | SP_71772 | 7076nt | ORF1: Hel – RdRp1 ORF2: Dicistro_VP4 |
| S3_L001 | SP_36073 | 9601nt | ORF1: Hel – RdRp1 – RhV – Dicistro_vp4 – CRPV capsid |
| S3_L001 | SP_31066 | 9396 nt | ORF1: Hel – RdRp1 ORF2: RhV – [CRPV_capsid](https://www.genome.jp/dbget-bin/www_bget?pf:CRPV_capsid) |
| S5_L001 | SP_21627 | 9653 nt | ORF1: Hel – RdRp1 ORF2: RhV – [CRPV_capsid](https://www.genome.jp/dbget-bin/www_bget?pf:CRPV_capsid) |

ID: contig identification, bp: base pairs.

**Table S2.** Conserved motifs of Helicase.

| **Library code** | **Contig_ID** | Walker A  **(GxxGxGKS/T)** | Walker B  **(Qx_5_ DD)** | Walker C  **(Kkx_4_ Px_5c_NSN)** |
| --- | --- | --- | --- | --- |
| S5_L001 | SP_11226 | GPSSQGKT | INHFIIND | EKTWLEPELVTVTTN |
| S3_L001 | SP_44182 | GLSSQGKT | INHFIIND | EKTWLEPELVTVTTN |
| S5_L001 | SP_34331 | GPSSQGKT | INHFIIND | EKTWLEPELVTVTTN |
| S6_L001 | SP_40484 | ND | INHFIIND | EKTWLEPELVTVTTN |
| S5_L001 | SP_24176 | GGSSIGKS | SWAGVFDD | GKVFCNFQCVVFTSN |
| S22_L001 | SP_44506 | GGSSIGKS | SWAGVFDD | GKVFCNFQCVVFTSN |
| S3_L001 | SP_83441 | GGSSIGKS | SWAGVFDD | GKVFCNFQCVVFTSN |
| S22_L002 | SP_10981 | GGSSIGKS | SWAGVFDD | GKVFCNFQCVVFTSN |
| S3_L001 | SP_46102 | GGSSVGKT | QWGLIFDD | GKIFYNHSLIGITTN |
| S39_L002 | SP_44658 | GNSSIGKT | QWGLIFDD | GKIFHNQSLIGITTN |
| S31_L002 | SP_28427 | GDSGVGKS | TTGIILDD | AMTALEPMVVIGTTN |
| S31_L001 | SP_96915 | GDSGVGKS | TTGIILDD | AMTALEPMVVIGTTN |
| S3_L001 | SP_71772 | GGSSLGKS | VWFMVLDD | GKTPLRPKFVMGTTN |
| S3_L001 | SP_36073 | GSPGIGKS | QWSEIGAD | GTKYVSLECVIVDTN |
| S3_L001 | SP_31066 | GGTSVGKS | QRYILFDD | GTQCITSAALIATSN |
| S5_L001 | SP_21627 | GGTSVGKS | QRYILFDD | GTQCITSAALIATSN |

| ND: not detected |  |  |
| --- | --- | --- |

**Table S3.** Conserved motifs of Protease.

| **Library code** | **Contig_ID** | **GxCG** | GXHXXG |
| --- | --- | --- | --- |
| S5_L001 | SP_11226 | GLCG | GLHLGG |
| S3_L001 | SP_44182 | GLCG | GLHLGG |
| S5_L001 | SP_34331 | GLCG | GLHLGG |
| S6_L001 | SP_40484 | GLCG | GLHLGG |
| S5_L001 | SP_24176 | GDCG | GIHIAG |
| S22_L001 | SP_44506 | GDCG | GIHIAG |
| S3_L001 | SP_83441 | GDCG | GIHIAG |
| S22_L002 | SP_10981 | GDCG | GIHIAG |

**
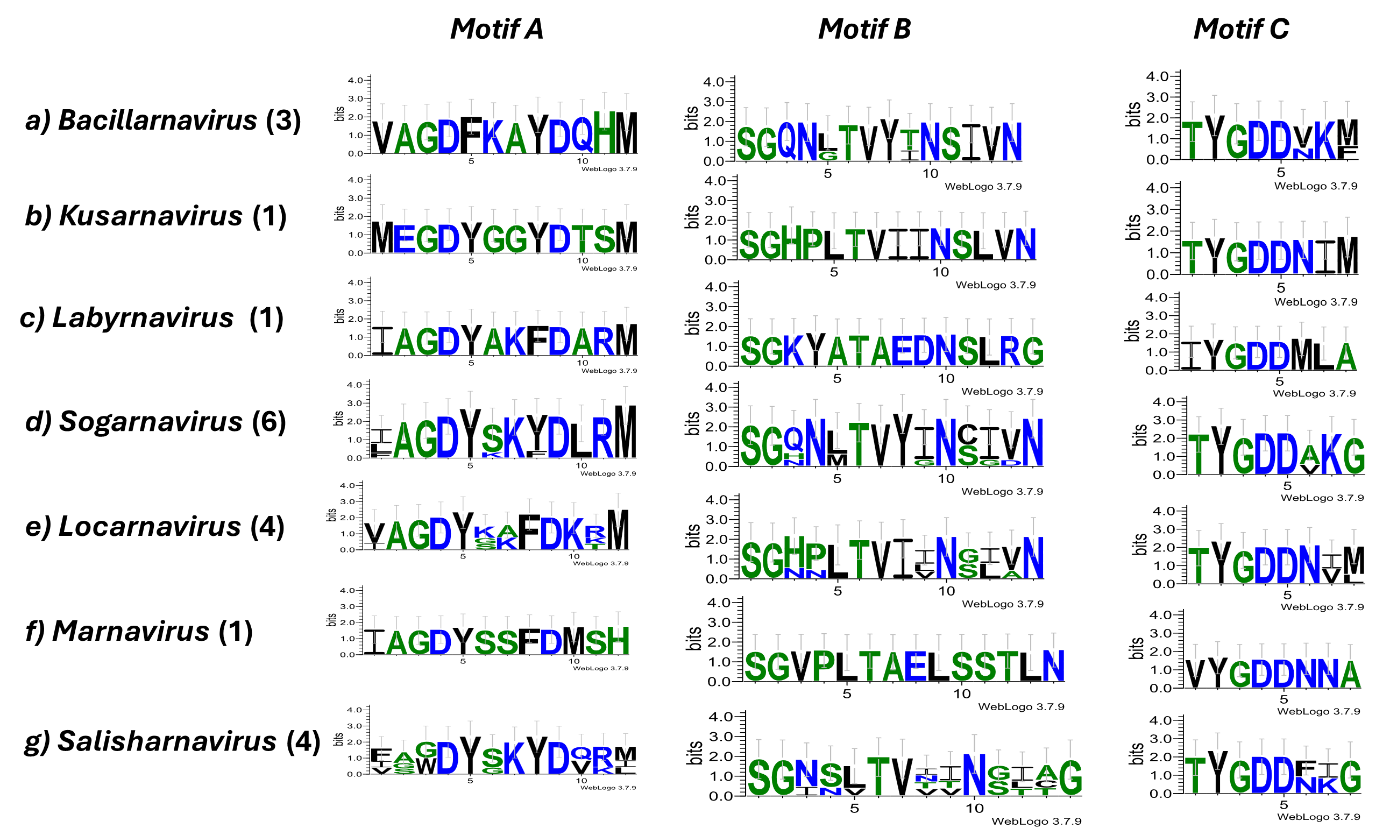
**

**Figure S1.** The RdRP motif sequences of members of the genus *Marnaviridae* logos generated in Weblogo 3**.**


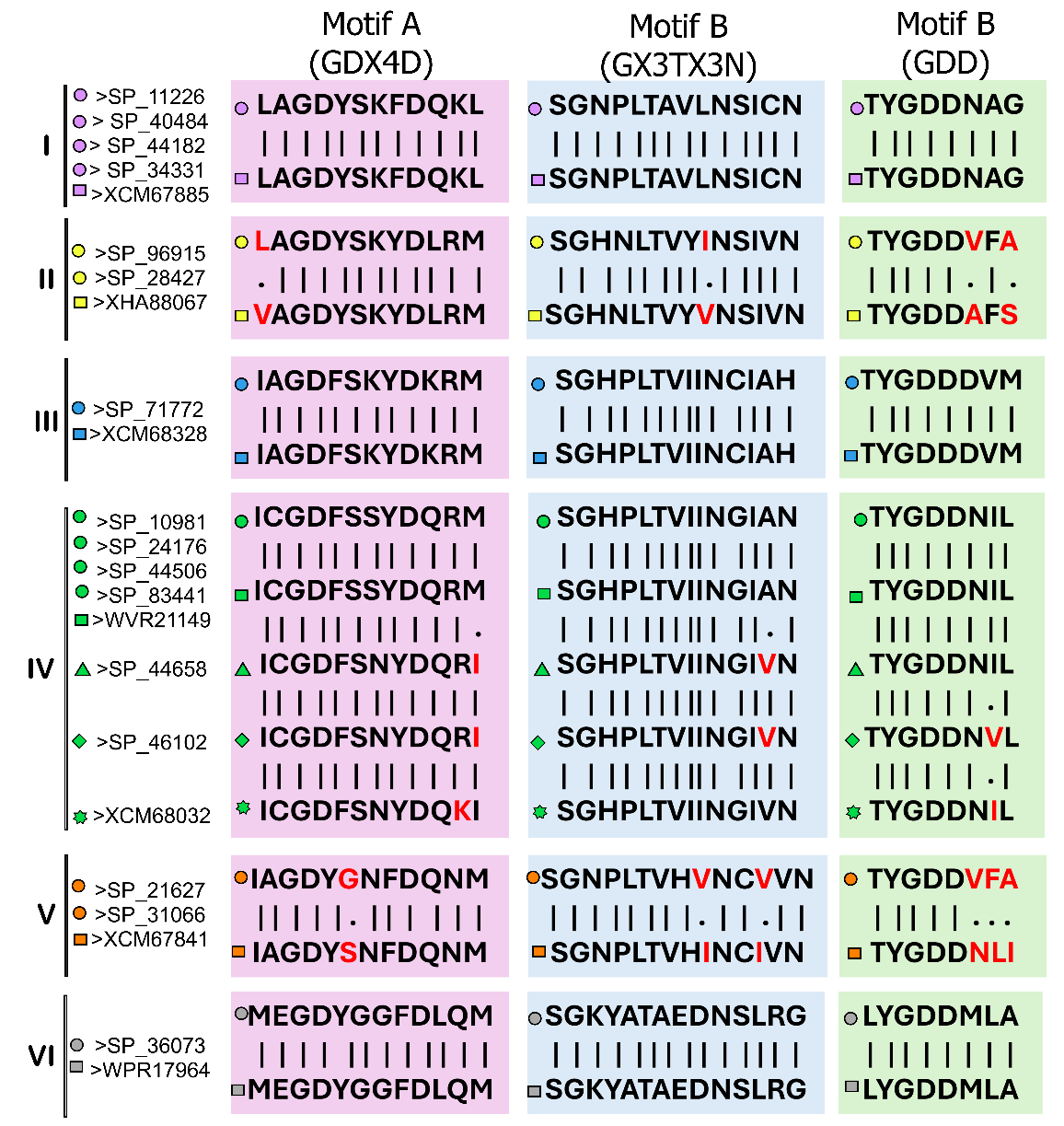


**Figure S2.** **Comparison of the reasons predicted in the RdRp domain of the viruses identified with the best BLASTx best-hit.** Motif A (purple), motif B (blue) and motif C (green) are highlighted. Symbols (circle, square, triangle, diamond, and star) indicate matches between detected motif sequences. Sequences identified in the study are shown with colored circles, except SP_44658 (triangle) and SP_46102 (diamond). BLASTx references are highlighted by colored squares, except XCM68032 (star).


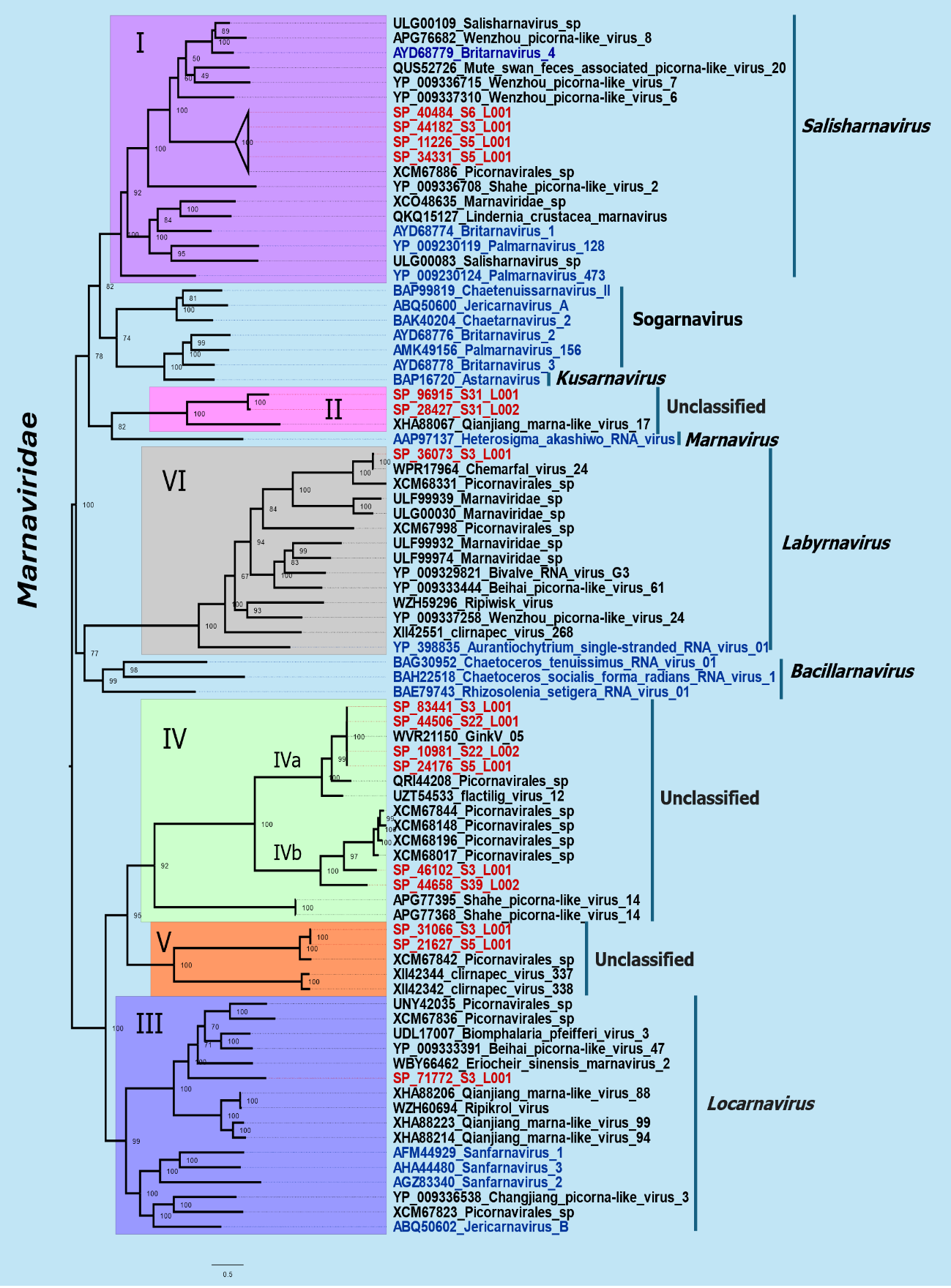


**Figure S3. Maximum likelihood phylogeny of the structural region of *Marnaviridae*.** Viruses identified in water samples are labeled with contig and library ID (red font), *Marnaviridae* members (blue font), and representative Picornavirales (brown font). The GenBank accession number is provided for each sequence, followed by the species or virus name. The tree was inferred using IQ-TREE v2.4.0, applying the LG+F+R6 substitution model with 1,000 bootstrap replicates. Bootstrap support values are displayed at each tree node.


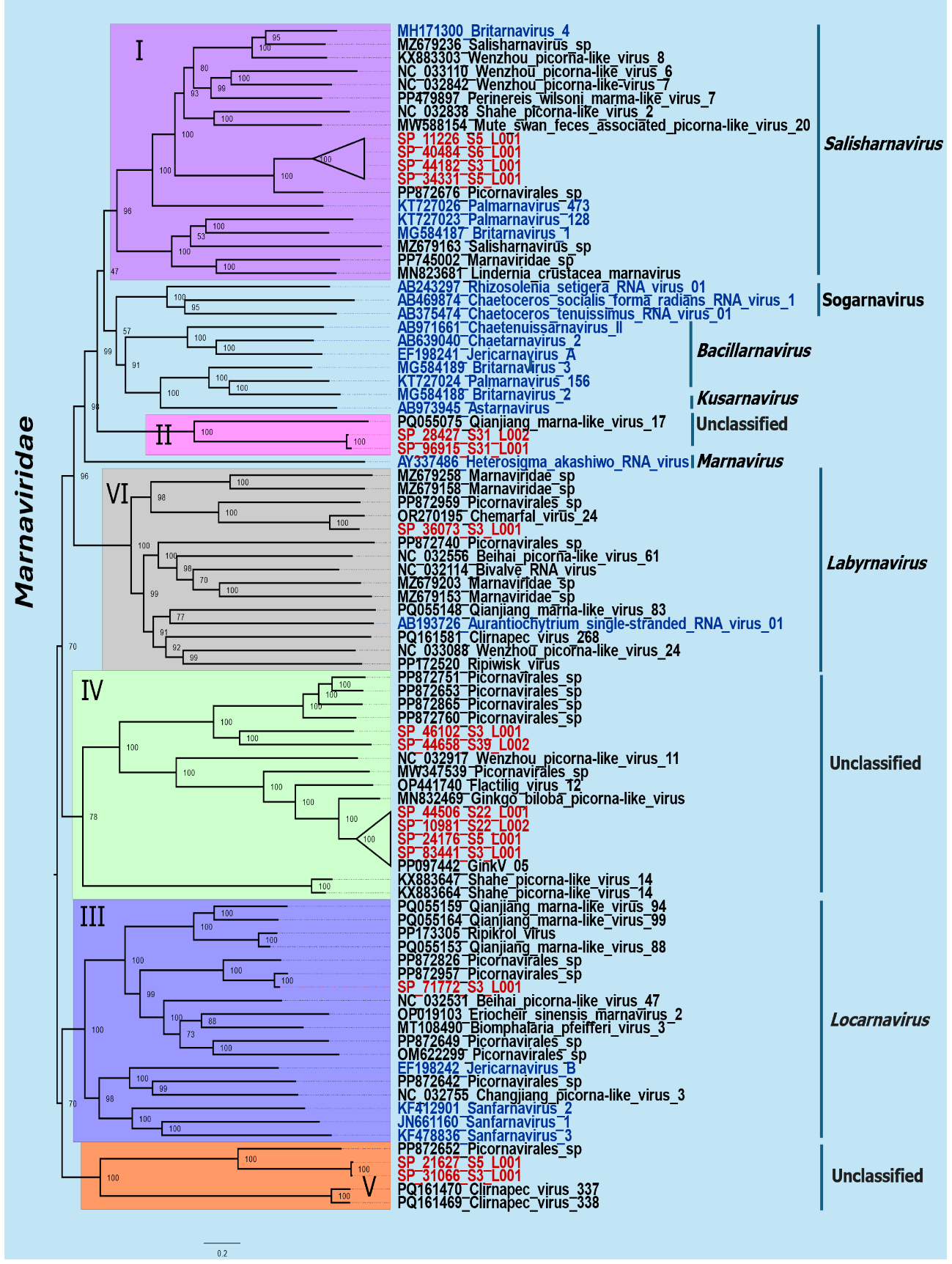


**Figure S4. Maximum likelihood nucleotide phylogeny of the *Marnaviridae* genome.** Viruses identified in water samples are labeled with contig and library ID (red font), *Marnaviridae* members (blue font), and representative Picornavirales (brown font). The GenBank accession number is provided for each sequence, followed by the species or virus name. The tree was inferred using IQ-TREE v2.4.0, applying the TVM+F+R6 substitution model with 1,000 bootstrap replicates. Bootstrap support values are displayed at each tree node.
